# Two-dimensional simulation of optical coherence tomography images

**DOI:** 10.1038/s41598-019-48498-2

**Published:** 2019-08-21

**Authors:** Thomas Brenner, Peter R. T. Munro, Benjamin Krüger, Alwin Kienle

**Affiliations:** 1 Institut für Lasertechnologien in der Medizin und Meßtechnik an der Universität Ulm, Ulm, 89081 Germany; 2 University College London, Department of Medical Physics and Biomedical Engineering, London, WC1E 6BT UK

**Keywords:** Imaging and sensing, Computational biophysics, Computational science

## Abstract

An algorithm for the simulation of two-dimensional spectral domain optical coherence tomography images based on Maxwell’s equations is presented. A recently developed and modified time-harmonic numerical solution of Maxwell’s equations is used to obtain scattered far fields for many wave numbers contained in the calculated spectrum. The interferometer setup with its lenses is included rigorously with Fresnel integrals and the Debye-Wolf integral. The implemented model is validated with an existing FDTD algorithm by comparing simulated tomograms of single and multiple cylindrical scatterers for perpendicular and parallel polarisation of the incident light. Tomograms are presented for different realisations of multiple cylindrical scatterers. Furthermore, simulated tomograms of a ziggurat-shaped scatterer and of dentin slabs, with varying scatterer concentrations, are investigated. It is shown that the tomograms do not represent the physical structures present within the sample.

## Introduction

Optical coherence tomography (OCT) is an imaging modality that enables the recording of high-resolution depth profiles of semi-transparent scattering media^1–3^. The depth profiles are obtained using a broadband interferometer setup by interfering electromagnetic fields from a reference arm and a sample arm. The technique is used in many fields such as ophthalmology^4^, dermatology^5–7^, intravascular imaging^8,9^, and art conservation^10–12^. It is expected that the global OCT market will reach US$1.8 billion by 2024^13^. On the experimental side, several innovations have led to improved imaging resolution^14^, imaging speed^15^ and working distance^16,17^. On the theoretical side, however, the link between microscopic properties of the scattering medium and the resulting tomograms is not yet fully understood^18–21^. It is desireable to link the microscopic properties of the sample to features of the recorded tomograms, to reduce interpretation errors due to artifacts and to improve existing imaging devices. Since calculating the light scattered by an arbitrary sample geometry is complex, computer simulations are employed in many cases. Many computer simulations of OCT images use a Monte Carlo model (see for example^22–25^) which neglects the wave nature of light, meaning that phenomena such as diffraction and coherence are also neglected. There are models which use the extended Huygens-Fresnel principle, meaning that the investigated sample is treated as a turbid medium where the refractive index exhibits random spatial fluctuations^26–28^. Full-wave approaches which rigorously consider Maxwell’s equations and include the interferometer configuration are often necessary in order to model image formation in OCT accurately. For example, the simulation of OCT imaging of blood cells requires the use of Maxwell’s equations^29^. There are simulations of OCT images of lung tissue^30,31^ where the interferometer setup has not been taken into account. Simulations of single microspheres have successfully been compared to experimental OCT data^32^. There are several proceedings dealing with OCT simulations based on Maxwell’s equations^33–35^. However, calculations including Maxwell’s equations for both the interferometer setup and the interaction of the electromagnetic field with the investigated media for large volumes are computationally expensive and only few works exist^36,37^.

In this study, a modified version of the algorithm presented in^38^ is used to simulate two-dimensional OCT tomograms. Our previous work^39^ is extended to complex scatterers. The interferometer setup is included rigorously by using Maxwell’s equations. The structure of this study is as follows: First, the derivation of the OCT algorithm is presented and both the resulting incident beam and the achieved imaging depth are discussed. In the second section, tomograms of different scatterers are presented. Both single and multiple cylindrical scatterers, a ziggurat-shaped scatterer and dentin models are investigated. The results for single and multiple cylindrical scatterers are validated by comparing them to tomograms calculated with a finite-difference time-domain (FDTD) version of the algorithm described in^36,37^ and the features of the simulated OCT images are discussed. By going from single to multiple cylindrical scatterers and then onwards to a more realistic scatterer model for tubules in dentin, it is shown for both simple cases and more involved cases that the resulting tomograms do not represent the exact geometrical shapes.

## Theory

**Figure 1:**
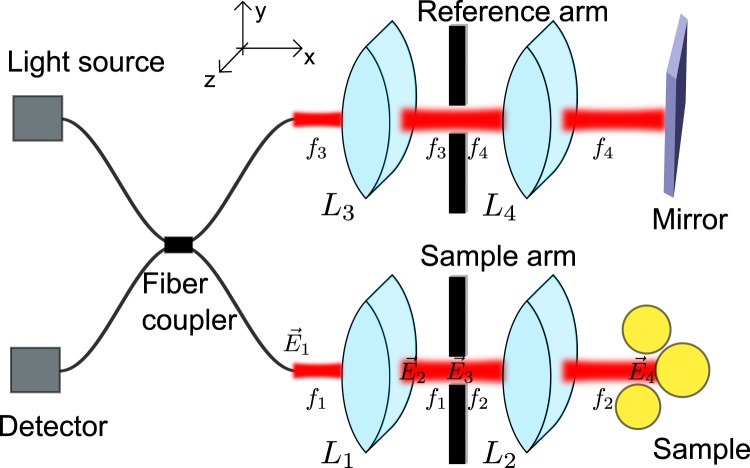
Schematic of the simulated OCT setup. Lenses *L*_1_ and *L*_3_ have a focal length of *f*_1,3_ = 25 mm while lenses *L*_2_ and *L*_4_ have a focal length of *f*_2,4_ = 36 mm. The radius of the aperture placed between the lenses is *h* = 3.5 mm. The light source has a central wavelength of *λ*_0_ = 1.3 *μ*m and a FWHM of Δ*λ* = 170 nm. A fiber coupler splits and recollects the light so that the detector records interference fringes containing the depth information. In spectral domain OCT, the mirror in the reference arm does not need to be moved and a spectrometer detects a wave-number-dependent intensity. The model used corresponds closely to the setup of the “Thorlabs Telesto II”^59^.

Optical coherence tomography uses the interference between a reference arm and a sample arm signal in order to reconstruct the relative phase of the light. In order to develop a realistic algorithm for an OCT setup based on Maxwell’s equations, the electromagnetic fields need to be propagated through the optical system. Note that the model for the interferometer setup presented here uses a similar approach as that derived in^36,37^. Since the computational effort is considerable, we restrict ourselves to two-dimensional simulations. Figure 1 shows the model and its calculation steps: Light from a broadband light source is coupled out of an illumination fiber. The fields are split so that both the reference mirror and the sample are illuminated. The fields propagate through *L*_1_ and *L*_2_ in the sample arm and *L*_3_ and *L*_4_ in the reference arm, respectively. For arbitrary two-dimensional scatterers, a numerical solution of Maxwell’s equation is employed in order to obtain the scattered fields in the sample arm. The scattered fields are propagated back through the lens system and coupled back into the detection fiber, for each wave number, interfering with the reference fields. This means that illumination and detection is modeled as a superposition of coherent modes which closely resembles a swept-source setup. However, since the light in the reference arm and the sample arm comes from the same source, and since only the correlation between the reference and the sample arm is measured in OCT, the calculation is mathematically equivalent to a spectral domain setup with a spectrally broad light source^37,40^. In the following, a description of each calculation step of the OCT model is given.

Assuming a weakly guiding fiber that emits a wave field, and omitting a time dependence of ${e}^{-i\omega t}$, the field ${\overrightarrow{E}}_{1}$ exiting the fiber can be written as

1 $${\overrightarrow{E}}_{1}={E}_{0}{e}^{-\frac{{y}^{2}}{{w}_{0}^{2}}}\hat{p},$$

where $\hat{p}$ indicates either polarisation in *y*-direction or in *z*-direction, while propagation is in *x*-direction as indicated in Fig. 1. *w*_0_ is half of the fiber’s mode field diameter, taken to be *w*_0_ = 4.6 *μ*m in the given setup. *E*_0_ is the maximum magnitude of the field. In the following, the vectorial character $\hat{p}$ of the fields is omitted since the in-plane component and the out-of-plane component may be treated independently of each other, since they do not mix in such a two-dimensional geometry. Using a two-dimensional angular spectrum of plane waves approach, the field incident on *L*_1_ can be calculated as

2 $${E}_{2}=\frac{1}{\sqrt{2\pi }}\frac{{E}_{0}{w}_{0}}{\sqrt{2}}\,{\int }_{{k}_{y}=-k}^{k}\,{e}^{-\frac{1}{4}{k}_{y}^{2}{w}_{0}^{2}+i{k}_{y}y+i\sqrt{{k}^{2}-{k}_{y}^{2}}x}d{k}_{y},$$

where *k* is the wave number and *k*_*y*_ is its component in *y*-direction. Setting $x={f}_{1}$ since the incident field is sampled on the first lens *L*_1_ (or *L*_3_) and using the paraxial approximation to write $\sqrt{{k}^{2}-{k}_{y}^{2}}\approx k-\frac{{k}_{y}^{2}}{2k}$ for ${k}_{y}\ll k$, the field incident on *L*_1_ is given by

3 $${E}_{2}\approx \frac{{E}_{0}{w}_{0}}{\sqrt{\frac{2i{f}_{1}}{k}+{w}_{0}^{2}}}{e}^{ik{f}_{1}-\frac{k{y}^{2}}{2i{f}_{1}+k{w}_{0}^{2}}},$$

where *f*_1_ is the focal length of the first lens *L*_1_. For the reference arm, the same expression can be used, especially when ${f}_{1}={f}_{3}$, since ideally, the system is perfectly matched. In order to propagate the field through the lens towards the focal plane common to both *L*_1_ and *L*_2_ (or *L*_3_ and *L*_4_), a two-dimensional Fresnel integral is used together with the paraxial transmission function of the lens $t={e}^{-\frac{-ik}{2{f}_{1}}{y}^{2}+ik{n}_{L1}{D}_{L1}}$^41^ where *n*_*L*1_ is the refractive index of the lens *L*_1_ and *D*_*L*1_ is its maximum thickness at the center. The two-dimensional Huygens-Fresnel integral is given as^42^

4 $$U(y)={e}^{\frac{-i\pi }{4}}\,{\int }_{-\infty }^{\infty }\,\frac{1}{\sqrt{\lambda r}}{U}_{inc}(\xi )\,\cos (\frac{\theta }{2}){e}^{ikr}d\xi $$

where *θ* is the angle belonging to a point between the *x*-axis and the *y*-axis, $\xi$ is a lateral point on the input plane and *U*_*inc*_ is the incident field at the input plane. *λ* is the wavelength. Similarly to the three-dimensional approximations for propagation through lenses in^41^, the distance $r=x\sqrt{1+{(\frac{y-\xi }{x})}^{2}}$ in two dimensions is approximated as $r\approx {f}_{1}$ in the amplitude factors and to second order as $r\approx {f}_{1}(1+\frac{1}{2}{(\frac{y-\xi }{{f}_{1}})}^{2})$ for the argument of the phase factor. The cosine is approximated as $\cos \,(\frac{\theta }{2})\approx 1$ for small angles. The field between the lenses *L*_1_ and *L*_2_ (*L*_3_ and *L*_4_) in paraxial approximation at the focus becomes

5 $$\begin{array}{ccccc}{E}_{3} & = & {\int }_{{\rm{\infty }}}^{{\rm{\infty }}}\,\frac{{e}^{-i\frac{\pi }{4}}}{\sqrt{\lambda {f}_{1}}}{E}_{2}(\xi )t(\xi ){e}^{ik{f}_{1}[1+\frac{1}{2}{(\frac{y-\xi }{{f}_{1}})}^{2}]}d\xi  & = & (1-i){w}_{0}{E}_{0}\frac{\sqrt{\pi }}{\sqrt{2{f}_{1}\lambda }}{e}^{2ik{f}_{1}+ik{D}_{L1}{n}_{L1}-{(\frac{yk{w}_{0}}{2{f}_{1}})}^{2}}.\end{array}$$

The thickness of the lens *D*_*L*_ can be neglected. The paraxial approximation is valid here since OCT uses a small numerical aperture. In many imaging systems such as OCT, an afocal and telecentric lens setup with the aperture stop between *L*_1_ and *L*_2_ (*L*_3_ and *L*_4_) is used. The aperture stop appears then at infinity from both the sample space and the detector space. For this reason, the Debye-Wolf formalism can be used^43–45^ which represents a rigorous solution of Maxwell’s equations. As described above, the scalar Fresnel integral is sufficient to calculate the propagation of the fields through a lens system with a low NA as is used in OCT systems. However, a rigorous solution of Maxwell’s equations is needed in order to couple the fields into the simulation grid of the numerical solver^38^. For this reason, the Debye-Wolf integral is employed in order to calculate the electromagnetic field in the sample space. In three dimensions, the Debye-Wolf integral is given by^43,44,46,47^

6 $$\overrightarrow{E}(\rho ,\varphi ,z)=-\,\frac{ikf}{2\pi }{e}^{ikf}\,{\int }_{\varphi {\rm{^{\prime} }}=0}^{2\pi }\,{\int }_{0}^{\alpha }\,\overrightarrow{e}(\theta ,\varphi {\rm{^{\prime} }}){e}^{ik\rho \sin (\theta )\cos (\varphi {\rm{^{\prime} }}-\varphi )+ikz\cos (\theta )}\,\sin (\theta )d\theta d\varphi {\rm{^{\prime} }}.$$

Here, $\rho =\sqrt{{x}^{2}+{y}^{2}}$ is the radial coordinate where the field $\overrightarrow{E}(\rho ,\varphi ,z)$ is calculated while *z* is the distance from the focal position. $\varphi$ is the angle of the polar coordinate system and $\overrightarrow{e}(\theta ,\varphi {\rm{^{\prime} }})=\sqrt{\cos \,\theta }R-1(\varphi {\rm{^{\prime} }})L(\theta )R(\varphi {\rm{^{\prime} }}){\overrightarrow{E}}_{inc}(\theta ,\varphi {\rm{^{\prime} }})$ is the field, refracted by the lens, on a Gaussian reference sphere centered on the geometrical focus of *L*_2_ (and *L*_4_)^48,49^. Note that many authors omit the global phase *e*^*ikf*^. In three dimensions, the rotation matrix $$R(\varphi {\rm{^{\prime} }})=(\begin{array}{ccc}\cos \,\varphi {\rm{^{\prime} }} & \sin \,\varphi {\rm{^{\prime} }} & 0\\ -\sin \,\varphi {\rm{^{\prime} }} & \cos \,\varphi {\rm{^{\prime} }} & 0\\ 0 & 0 & 1\end{array})$$ is used to decompose field vectors into components which are parallel and perpendicular to the plane of refraction. Refraction by the lens is represented by the rotation matrix *L* which is given by $$L(\theta )=(\begin{array}{ccc}\cos \,\theta  & 0 & \sin \,\theta \\ 0 & 1 & 0\\ -\sin \,\theta  & 0 & \cos \,\theta \end{array}).$$ Since we wish to reduce the computational effort by describing a two-dimensional OCT-system, the Debye-Wolf integral for cylindrical lenses is used. The integration is performed over one angle *θ* and $\varphi ^{\prime} =0$. It can be seen immediately that the rotation matrix $R(\varphi ^{\prime} =0)$ becomes an identity matrix since no rotation needs to be applied and the expression sin *θ* stemming from the Jacobian element from the conversion from Cartesian to spherical coordinates disappears in cylindrical coordinates. In order to determine the prefactors for the cylindrical Debye-Wolf integral, we follow the derivations in^46^: The two-dimensional Green’s function is given as

7 $$G(p,x)=\frac{i}{4}{H}_{0}^{(1)}(k|\overrightarrow{p}-\overrightarrow{x}|)\approx \frac{i}{4}\sqrt{\frac{2}{\pi kR}}{e}^{ik(R-\hat{q}\cdot \overrightarrow{x})}{e}^{-i\frac{\pi }{4}},$$

where *R* is the radius of the reference circle, $\overrightarrow{p}$ is a point on the wave front and $\hat{q}$ is a unit vector from the focus position to the point on the wavefront indicated by $\overrightarrow{p}$. The gradient of the Green’s function is given as

8 $$\nabla G(p,x)={e}^{-i\frac{\pi }{4}+ik(R-\hat{q}\cdot \overrightarrow{x})}\sqrt{\frac{k}{8\pi R}}\hat{n}.$$

Both the Green’s function and its gradient can be used in the vectorial Kirchoff-Fresnel diffraction integral. Following the derivation in^46^ with the developed two-dimensional approach, the Debye-Wolf integral is obtained as

9 $$E=\sqrt{\frac{k}{2\pi R}}{e}^{-i\frac{\pi }{4}}\,{\int }^{}\,{e}^{ik(R-\hat{q}\cdot \overrightarrow{x})}d\sigma =\sqrt{\frac{kR}{2\pi }}{e}^{-i\frac{\pi }{4}}\,{\int }^{}\,{e}^{ik(R-\hat{q}\cdot \overrightarrow{x})}d{\rm{\Gamma }},$$

where the last equality is valid when no aberrations are present so that the surface converging to the focal point is cylindrical ($d\sigma =Rd{\rm{\Gamma }}$). It is noted here that the one-dimensional Debye-Wolf integral is also described by Sheppard^50^. By using the one-dimensional Debye-Wolf integral, the aperture is assumed to be a slit and the lenses are approximated as cylindrical lenses. Sheppard^50^ gives comparisons of the two-dimensional (cylindrical lens) and the three-dimensional case (spherical lens) and states that “The general features of both the cylindrical and spherical cases are similar …” which gives an idea of what the approximation of a three-dimensional interferometric system as two-dimensional encompasses. Continuing therefore with the one-dimensional Debye-Wolf integral with lenses fulfilling the sine condition in a coordinate system as shown in Fig. 1, one obtains

10 $${\overrightarrow{E}}_{4}=\sqrt{\frac{{f}_{2}}{\lambda }}{e}^{-i\frac{\pi }{4}+ik{f}_{2}}\,{\int }_{-\alpha }^{\alpha }\,\sqrt{\cos \,\theta }(\begin{array}{c}{E}_{x}\,\cos \,\theta -\,\sin \,\theta {E}_{y}\\ {E}_{x}\,\sin \,\theta +\,\cos \,\theta {E}_{y}\\ {E}_{z}\end{array}){e}^{ik(y\sin \theta +x\cos \theta )}d\theta .$$

Note how the Debye-Wolf integral, differing from a scalar approach, gives rise to light with a component of polarisation parallel to the optical axis. If the NA of the optical system is small, however, the change in polarisation is not significant^45^. Identifying ${E}_{x}={E}_{y}=0$ and ${E}_{z}={E}_{3}$ for perpendicular polarisation of the incident light and ${E}_{x}={E}_{z}=0$ and ${E}_{y}={E}_{3}$ for parallel polarisation, the incident field can now be computed.

**Figure 2:**
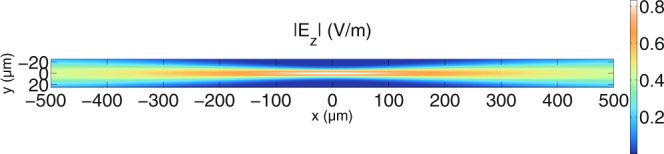
Plot of the incident focused beam for perpendicular polarisation for *λ* = 1300 nm in a medium of *n*_*med*_ = 1.35. This is the shape of the incident beam when no scatterers are present. The beam arises from numerical integration of the complex input amplitudes given to the Debye-Wolf integral. Note that in the limits of the Debye-Wolf integral determined by the NA, the refractive index of the medium is set to 1 in order to avoid strong side lobes.

Having propagated the field from the fiber end face to the plane between the lenses with the Fresnel integral and from the middle plane to the focal region of the second lens with the Debye-Wolf integral, the propagated fields impinge now either on a scatterer (pathway through *L*_1_ and *L*_2_) or on a reference mirror (*L*_3_ and *L*_4_). For simplicity and because a good optical system is well-aligned, we assume that the reference and the sample arm have the same fields in their focal regions, stemming from similar lenses. However, different lenses for the reference and for the sample arm can easily be considered and the derivation leading to equation (9) shows that lens aberrations can readily be included in the simulations. For the sample arm, the field described by equation (10) can be coupled into the simulation grid of the solver, knowing that the integration is performed over a modified angular spectrum of plane waves. It would exceed the scope of this paper to describe the numerical solver in detail. It suffices to say that as described in^38^, the solver gives the near fields and far fields obeying Maxwell’s equations for a certain scatterer distribution for an indicated wavelength *λ* or wave number *k*. In the following, the aforementioned solver is referred to as the MAXWELL-solver. Since the scattered fields collected by the lens *L*_2_ are at distances that are of the order of millimeters due to the focal length *f*_2_, we sample the far fields on a circle in front of the lens, propagate the fields back with the Fresnel integral used in equation (5) and we propagate the fields from the mid plane back to the fiber with the Debye-Wolf integral used in equation (10). Figure 2 shows the absolute value of the incident beam in the simulation grid. In all two-dimensional color-encoded plots, the Haxby colormap is used^51,52^.

### Reference arm

In principle, the MAXWELL-solver could calculate a far field backscattered from a mirror set up in the reference arm. However, in order to save calculation time, the far fields stemming from the mirror are calculated analytically. It is shown in the following that the Debye-Wolf integral can be solved analytically in two dimensions in the limit of distances far from the focus. Since the focus is at a distance of millimeters away from the lens *L*_4_ and since the light needs to propagate back from the mirror to the lens, the condition of large distances (far field condition) is fulfilled. First, the plane wave in equation (10) is rewritten in polar coordinates as

11 $${e}^{ik(y\sin \theta +x\cos \theta )}={e}^{ik(l\sin \alpha \sin \theta +l\cos \alpha \cos \theta )}={e}^{ikl\cos (\alpha -\theta )},$$

where *l* is the radius and *α* is the angle of the polar coordinates. Using the Jacobi-Anger expansion^53^, the expression can be written as

12 $${e}^{ikl\cos (\alpha -\theta )}=\mathop{\sum }\limits_{n=-{\rm{\infty }}}^{{\rm{\infty }}}\,{i}^{n}{J}_{n}^{(1)}(kl){e}^{in(\alpha -\theta )}=\mathop{\sum }\limits_{n=-{\rm{\infty }}}^{{\rm{\infty }}}\,{i}^{n}\frac{1}{2}({H}_{n}^{(1)}(kl)+{H}_{n}^{(2)}(kl)){e}^{in(\alpha -\theta )},$$

where ${J}_{n}^{(1)}$ are Bessel functions of the first kind and order *n* and ${H}_{n}^{(m)}$ are Hankel functions of the *m*-th kind and order *n*. Considering only outgoing waves with the Hankel function of the first kind and calculating the approximation of the Hankel function for large *l* gives

13 $$\mathop{\sum }\limits_{n=-\infty }^{\infty }\,{i}^{n}\frac{1}{2}{H}_{n}^{(1)}(kl){e}^{in(\alpha -\theta )}\approx \frac{1}{2}\sqrt{\frac{2}{\pi kl}}{e}^{ikl-i\frac{\pi }{4}}\,\mathop{\sum }\limits_{n=-\infty }^{\infty }\,{e}^{in(\alpha -\theta )}.$$

The sum in equation (13) is identified as a series representation of the delta-function for periodic functions. This means that the contribution of equation (10) to the far field is one cylindrical wave per far field angle. Introducing the Fresnel reflection coefficients ${r}_{\perp ,\parallel }(\alpha )$, for the reflected fields perpendicular and parallel to the scattering plane respectively^54^, the expression at the lens *L*_4_ in the reference arm becomes

14 $${\overrightarrow{E}}_{5}=\sqrt{\frac{{f}_{2}}{\lambda }}{e}^{-i\frac{\pi }{4}+ik{f}_{2}}\sqrt{\cos \,\alpha }(\begin{array}{c}{E}_{x}\,\cos \,\alpha -\,\sin \,\alpha {E}_{y}\\ {E}_{x}\,\sin \,\alpha +\,\cos \,\alpha {E}_{y}\\ {E}_{z}\end{array})\pi \sqrt{\frac{2}{\pi kl}}{e}^{ikl-i\frac{\pi }{4}},$$

where *l* is the radial distance measured from the focal point of the lens that coincides with the position of the mirror in the performed simulations.

### Detection

Both the field backscattered by the sample and the field reflected by the mirror are sampled at the first lens surface and propagated with the Fresnel integral (5) to the middle plane of the lens. Subsequently, the fields in front of the detection fiber are calculated with the Debye-Wolf integral. Since the fields emitted by the fiber have been described by a Gaussian shape, the amount of light coupled back into the fiber is

15 $$\alpha (k)={\int }^{}\,({E}_{7,s}(y,k)+{E}_{7,r}(y,k)){e}^{-\frac{{y}^{2}}{{w}_{0}^{2}}}dy={\alpha }_{sca}(k)+{\alpha }_{ref}(k),$$

where *E*_7,*s*_ and *E*_7,*r*_ are the sample and reference arm fields, respectively, and may refer to either the parallel or perpendicular field components. The very weak contribution of the polarisation parallel to the *x*-direction is neglected. Having calculated the interferogram and weighting it according to the spectral power distribution similar to the descriptions in^39^, the OCT signal is post-processed using the inverse Fourier transform. Instead of calculating the absolute square of equation (15), it is possible to calculate only the cross-correlation by using

16 $${I}_{OCT,cross}={ {\mathcal F} }^{-1}({\alpha }_{sca}(k){\alpha }_{ref}{(k)}^{\ast }).$$

This removes the autocorrelation and the DC-artifact.

### Maximum displayable depth

A very important point to address is the link between the sampling of wave numbers contained in the incident spectrum^39^, the calculation time necessary for a simulation, and the maximum distance *ct*_*max*_ from the focus that will be visible in the tomogram. If the number of sampled wave numbers, *N*, in a given interval between ${\omega }_{{\min }}$ and ${\omega }_{{\max }}$ is too low, the signals associated with high depths will reappear at low depths due to aliasing by the inverse discrete Fourier transform. In the literature, the maximum visible depth is estimated as^55^

17 $${d}_{{\max }}\approx \frac{\pi }{2\delta k}=c{t}_{{\max }},$$

where *δk* is the spacing between the sampled wave numbers. The factor of 2 in the denominator indicates that the light undergoes a roundtrip through the considered medium. Equation (17) can be rewritten as

18 $${t}_{{\max }}\approx \frac{1}{2{n}_{med}}\frac{\pi (N-1)}{{\omega }_{{\max }}-{\omega }_{{\min }}},$$

where *n*_*med*_ is the refractive index of the medium. This is valid if the maximum time element available is at the index $\frac{N}{2}$ when *N* elements are used. However, the last element is ambiguous in a Fourier transform as it can be interpreted as −$\frac{N}{2}$ or $\frac{N}{2}$^56,57^. In this work, we use elements ranging from −$\frac{N}{2}$ to $\frac{N}{2}-1$. In other words, for even input *N*, an FFT-routine produces one more negative data point. Therefore,

19 $${t}_{max}=\frac{1}{2{n}_{med}}(\frac{N}{2}-1)\frac{2\pi (N-1)}{N({\omega }_{max}-{\omega }_{min})}=\frac{1}{2{n}_{med}}(\frac{N}{2}+\frac{1}{N}-\frac{3}{2})\frac{\pi b}{2\sqrt{{\rm{l}}{\rm{n}}\,\frac{1}{\varepsilon }}}.$$

**Figure 3:**
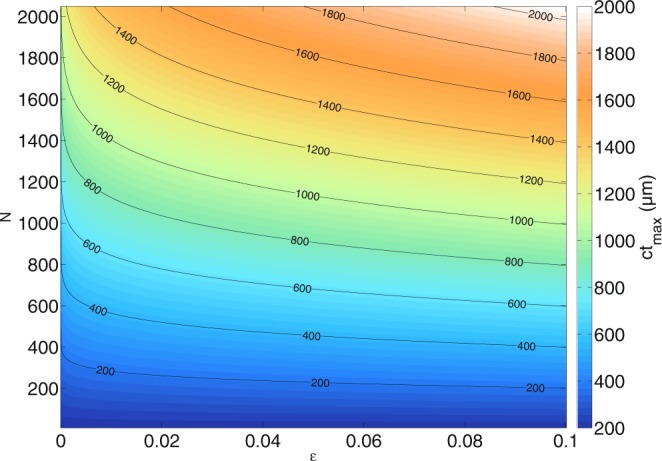
Dependency of the maximum tomogram depth on the number of wavelengths *N* calculated and the cut-off value $\varepsilon$ of the spectrum used^39^. The depth is counted from the focus position of the beam and it is given in *μ*m. The maximum depth is calculated for the simulated light source with *λ*_0_ = 1300 nm and Δ*λ* = 170 nm (*b* = 1.8 · 10^−14^ s) in a medium with *n*_*med*_ = 1.35. In short, this figure shows how the number of sampling points increases the maximum imaging depth and ultimately, the computation time.

This depth is the maximum visible depth in the performed simulations, counted from the focus position of the incident beam. It can be seen that the maximum depth depends on the chosen cut-off value^39^ of the amplitude $\varepsilon$ where the incident spectrum $S=\frac{b}{\sqrt{2}}{e}^{-{(\tfrac{b}{2}(\omega -{\omega }_{0}))}^{2}}$ with ${\omega }_{0}=\frac{2\pi c}{{\lambda }_{0}}$ at ${\rm{\min }}(\omega )$ and ${\rm{\max }}(\omega )$ has decreased to $\varepsilon \,{\rm{\max }}(S(\omega ))$ (the sensitivity of the spectrometer), the temporal width $b=4\frac{\sqrt{\mathrm{ln}(2)}}{{\rm{\Delta }}\omega }=2\frac{\sqrt{\mathrm{ln}(2)}{\lambda }_{0}^{2}}{\pi c{\rm{\Delta }}\lambda }$ of the pulse which is given by the OCT system (mainly the light source), and the number of wave numbers that are detected (the number of channels in the spectrometer) or calculated (the number of wave numbers that are given to the numerical solver). Note that this depth limit is due to the properties of the discrete Fourier transform^58^, not due to scattering or absorption in the medium. Choosing $\varepsilon$ means choosing an amplitude where the spectrum does not significantly contribute to the tomogram anymore due to its low amplitude. The number of wavelengths has a larger impact on the imaging depth (compare equation (19)) and at the same time it affects the total calculation time. One thus needs to find a compromise between imaging depth, size of the calculation grid, width of the absorbing boundaries and the number of image channels, *N*_*y*_, on the one hand, and an acceptable computation time on the other hand. Figure 3 shows the maximum visible depth from the focus position for different $\varepsilon$ and *N* for the given OCT system. For $N=160$, $b=1.8\cdot {10}^{-14}\,{\rm{s}}$ and $\varepsilon ={10}^{-3}$ in a medium of ${n}_{med}=1.35$, a depth of *ct*_*max*_ ≈ 92 *μ*m is achieved. The calculation time for a cylindrical scatterer with *a* = 20 *μ*m radius and refractive index *n* = 1.42 with the aforementioned *N* wave numbers with *N*_*y*_ = 83 scan positions and a grid resolution of about $\frac{{\lambda }_{0}}{24}=\frac{1.3\cdot {10}^{-6}}{24}\,{\rm{m}}$ on an AMD EPYC 7451 24-Core processor (where each core hosts two threads running at 2.3 GHz) was about 144 hours at 12 threads with 1400 × 1400 grid cells.

## Results

In the following, selected tomograms are presented in order to give an overview of the effects that appear in OCT imaging simulated with Maxwell’s equations in two dimensions. Similar to^59^, we consider an imaging system with ${f}_{1}=25\,{\rm{m}}{\rm{m}}$, ${f}_{2}=36\,{\rm{m}}{\rm{m}}$ and an aperture radius of $h=3.5\,{\rm{mm}}$. The central wavelength is *λ*_0_ = 1.3 *μ*m and we use a FWHM of Δ*λ* = 170 nm (related to the maximum amplitude) in all presented simulations. The OCT signals are calculated with equation (16). It is noted here that the results are sensitive to the parameters of the setup. For example, tomograms computed for a central wavelength of *λ*_0_ = 845 nm (see Supplementary Fig. S1) may have a different structure compared to tomograms computed with *λ*_0_ = 1.3 *μ*m. The absorbing boundaries of the grid have a width of 10 *μ*m in every direction in all simulations, unless noted otherwise. In order to obtain tomograms, the incident focused beam is shifted along the lateral *y*-coordinate relative to the scatterer. The recorded spectrum is Fourier-transformed for each scanned position. The depth positions are calculated from the obtained time values *t* as $x=\frac{tc}{2{n}_{med}}$, where the factor of 2 accounts for the round trip of the light and the division by the refractive index *n*_*med*_ accounts for the increase of the optical path length due to the presence of a medium. We use subsampling (10 × 10 sub-cells per grid cell)^38^ in the simulation grid to reduce artifacts caused by the finite size of the grid cells on the scatterer boundary. Compared to previous work^39^, the new simulations include rigorously the interferometer setup and the phase shift due to the detection fiber being at another position in lateral direction when the beam is scanned over the sample. In OCT, scanning over the surface of a sample is done by moving the position of the illumination and detection fiber or by deflecting mirrors. This means that the scanned beam would impinge on the lenses at another position in *y*. However, as long as this deviation is small, especially since a good interferometer setup is designed to reduce such abberations, it is reasonable to assume that the beam still propagates through the center of the lenses in the simulations, which proves to be a reasonable approximation. For this reason, scanning over the sample consists of either moving the sample relative to the imaging system or moving the imaging system relative to the scatterer, yielding equivalent results.

### Small cylinder

**Figure 4:**
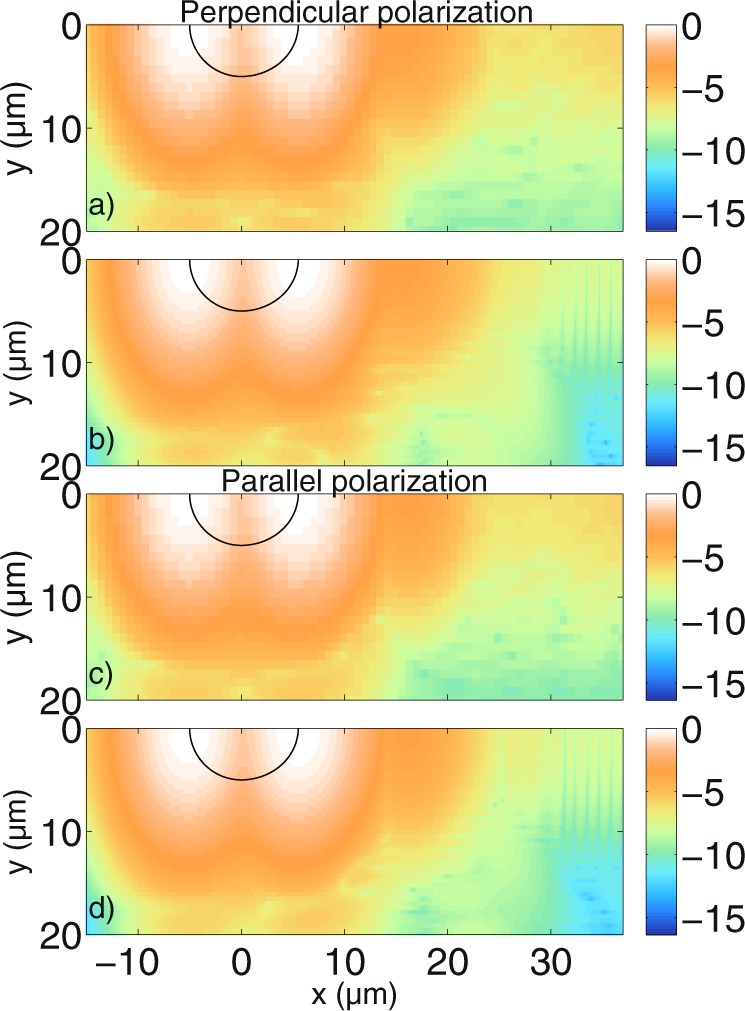
Comparison between tomograms simulated for (**a**,**b**) perpendicular polarisation and (**c**,**d**) parallel polarisation for an infinitely long cylindrical scatterer with *a* = 5 *μ*m radius. The scatterer, distorted according to the refractive index ratio, is indicated by a black line. (**a**,**c**) Are calculated with the MAXWELL-solver, while (**b**,**d**) are calculated with the FDTD-based model. With the MAXWELL-code, $N=160$ wave numbers and ${N}_{y}=83$ scan positions have been calculated, of which 42 are shown. The cut-off value is $\varepsilon ={10}^{-3}$ for the spectrum. The FDTD code evaluated $N=2048$ wave numbers and ${N}_{y}=50$ scan positions. Grid resolutions of $\tfrac{{\lambda }_{0}}{13}$ and $\tfrac{{\lambda }_{0}}{30}$ were employed by the MAXWELL-code and FDTD-code based models, respectively. The thickness of the boundaries in the numerical grid of the MAXWELL-solver were 40 *μ*m in each direction. The displayed values are the decadic logarithm of the normalized values of equation (16). The integrated error is 0.012 and 0.013 for perpendicular and parallel polarisation, respectively, indicating good agreement between the simulations.

We extend the investigation of simple cylindrical scatterers that has been described in previous work^39^. In order to validate the developed algorithm, the tomograms calculated using a modified version of the MAXWELL-solver and the interferometer setup described in the theory section are compared with the two-dimensional version of the FDTD-based code presented in^36,37^. Figure 4 shows the cross-correlation signal calculated with the absolute value of equation (16) for a cylindrical scatterer with a radius of *a* = 5 *μ*m and a refractive index of *n*_*cyl*_ = 1.42 in a medium of *n*_*med*_ = 1.35, which is similar to biological tissue in an organic solution^60,61^. The light is incident from the negative *x*-axis. Figure 4(a) shows the tomogram for incident light polarised perpendicular to the scattering plane (the x-y-plane in Fig. 1, noting that the polarisations do not mix in two dimensions). Figure 4(b) shows the same tomogram calculated with the algorithm presented in^37^. Figure 4(c) shows the tomogram for parallel polarisation and Fig. 4(d) shows the corresponding results obtained with the FDTD-based model^37^. Since the tomograms are symmetric, only half of the scanning positions are shown. Furthermore, the agreement between the simulations is quantified by calculating an integrated error over all image points as

$$\frac{\sqrt{{\sum }_{i,j}\,|{I}_{MAXWELL}-{I}_{FDTD}{|}^{2}}}{\sqrt{{\sum }_{i,j}\,|{I}_{MAXWELL}{|}^{2}}},$$

where *I*_*MAXWELL*_ and *I*_*FDTD*_ are the absolute values of the OCT signal amplitude for MAXWELL- and FDTD-based model, respectively, and the sum extends over rows and columns of the image. In order to do this, the result obtained with FDTD is interpolated to match the number of image points obtained with the MAXWELL-solver. It can be seen that the tomograms show good qualitative agreement. As described in^39^, the distance between the cylinder front and back side is *d* = 2*a*$\tfrac{{n}_{cyl}}{{n}_{med}}$ ≈ 10.52 *μ*m in Fig. 4. Note that all distances are relative optical distances that depend on the refractive indices of the medium and the scatterer. The signal behind the cylinder stems from multiple reflections inside the cylinder (transmission, 3 × reflection, transmission). Note that the resolution of the tomograms in Fig. 4(a,c) is lower than the resolution in Fig. 4(b,d). The computed tomograms show that the curvature of the smooth cylinder surface cannot be seen. Compared to our previous work^39^, refractive indices closer to the case of biological tissue have been used and the interferometer setup has been included rigorously according to Maxwell’s equations. With the obtained results, it is possible to explain the structure of experimental tomogram images presented elsewhere^62^.

### Larger cylinder

**Figure 5:**
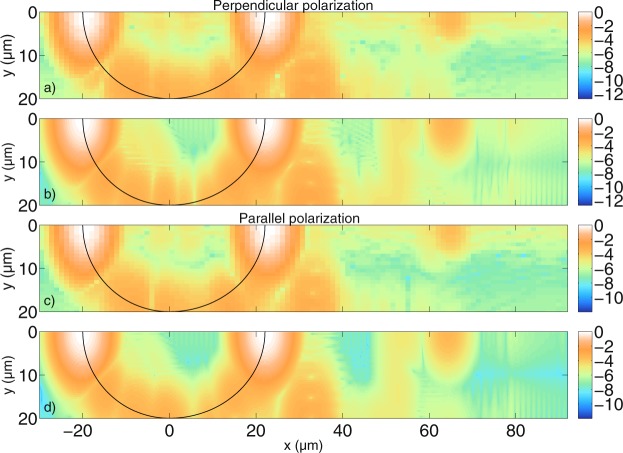
Comparison between tomograms simulated for (**a**,**b**) perpendicular polarisation and (**c**,**d**) parallel polarisation for an infinitely long cylindrical scatterer with radius *a* = 20 *μ*m. The cylinder, distorted by the refractive indices, is again indicated by a black line. (**a**,**c**) are calculated with the MAXWELL-solver while (**b**,**d**) are calculated with the FDTD-based model. With the MAXWELL-solver, ${N}_{y}=83$ scan positions have been calculated, of which 42 are visible. With the FDTD algorithm, ${N}_{y}=83$ scan positions have been calculated. The grid resolution used in the MAXWELL-solver is $\tfrac{{\lambda }_{0}}{24}$ and the width of the boundaries is 10 *μ*m in each direction. The other simulation parameters are the same as those in Fig. 4. Again, the integrated error indicates good agreement. For both polarisations, an error of 0.007 is obtained.

**Figure 6:**
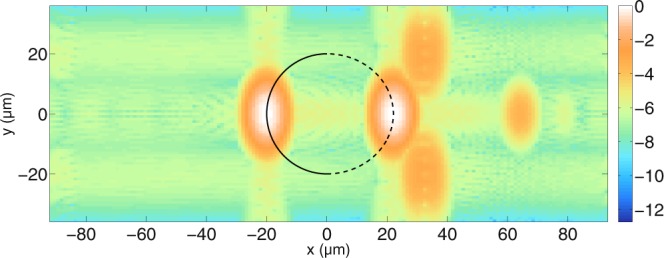
The full cylinder tomogram calculated with cylinder theory, plotted on a logarithmic scale for perpendicular polarisation. The simulation parameters are the same as those used in the MAXWELL-solver in Fig. 5. The black line indicates the cylinder side facing the scanning beam while the dotted line shows the distortion of the other side due to the refractive index of the cylinder.

The structure of the tomograms naturally changes when the cylinder size changes. In Fig. 5, simulations are presented for a cylindrical scatterer with radius *a* = 20 *μ*m, and with the same refractive indices as in Fig. 4. Again, Fig. 5(a,b) show the comparison of the perpendicular field component, while Fig. 5(c,d) show the parallel component. One can see that, analogously to Fig. 4, the distance between the cylinder front and back side is discernible as *d* = 2*a*$\tfrac{{n}_{cyl}}{{n}_{med}}$ ≈ 42.07 *μ*m. In the tomograms, the surface waves appear when the incident beam interacts with the cylinder at the lateral boundary (|*y*| ≈ *a*). Due to the low contrast between cylindrical scatterer and medium, the surface waves appear only at one depth position. Simulations with a higher refractive index contrast lead to more surface waves in the performed computations. As explained in the theory section, compared to our previous work^39^, different refractive indices have been used and the interferometer setup has been included rigorously. As a consequence, the fine substructure of the surface waves can be observed in the computed tomograms. To the best of our knowledge, this is the first time such a structure has been observed in computed tomograms of cylindrical scatterers. Whilst performing these simulations, we found that the structure of the surface waves is sensitive to the source condition that is used to generate the incident field. In particular, in the FDTD code, the full time-harmonic incident field should be calculated for every wave number *k* and then Fourier-transformed into time domain. Approximations to this approach that allow for an analytical expression of the incident field in time-domain in the FDTD code^63^, however, change the structure of the surface waves observed in the simulated tomograms. This is the first time, to our knowledge, that such an effect has been observed. At greater depths, signals from reflections inside the cylinder can be seen. Again, the comparison between MAXWELL-solver and the FDTD-based model show good agreement. However, the rippled structure that seems to trace the lateral side of the cylinder surface with an intensity that is orders of magnitude lower than the signals from the front and back of the cylinder differs between the simulations. Increasing the length of the absorbing boundaries in the MAXWELL-solver or changing the type of solver from the convergent Born series to a GMRES^64^ or a CGNR solver^65^ approach does not remove these structures. When the grid resolution is increased so that the size of the individual grid cells is smaller (and the curvature of the round structure is approximated better), it can be shown that the intensity of the rippled lateral structures tracing the cylinder curvature is reduced. In other words, it can be shown that these signals are artifacts from the numerical grid in both the time-harmonic MAXWELL-solver and the FDTD-based model. Another way to show this is to replace the numerical calculation with the analytical solution, by Bohren and Huffman^54^: Every plane wave incident at a certain angle gives rise to a scattered far field which can be weighted and integrated. Figure 6 shows the tomogram of a cylindrical scatterer calculated with the analytical solution, this time with both negative and positive *y*-values for clarity. The simulation shows that when there are no artifacts from a numerical grid, the rippled structures at the lateral cylinder sides disappear and the cylinder curvature cannot be seen. The black line shows where the cylinder surface facing the incident beam is located. The dotted black line shows where the other half of the cylindrical scatterer appears in the tomogram when the delay due to *n*_*cyl*_ along the path is taken into account. It can be seen that the scatterer appears distorted. Experiments with bubble raft phantoms and recorded OCT images^66,67^ show similar distortions. At a greater depth position behind the cylinder, at *x* = 63.75 *μ*m, the signal from multiple reflections inside the cylinder (transmission, 3 × reflection, transmission) can be seen clearly.

### Multiple cylinders

**Figure 7:**
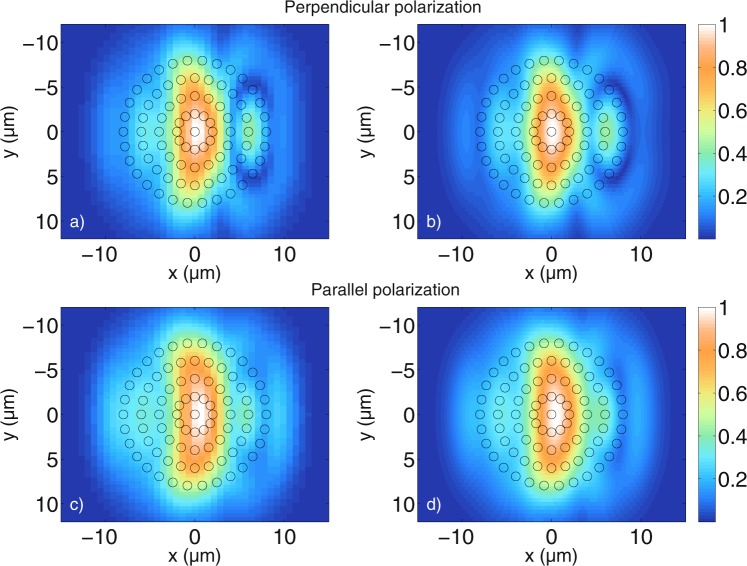
Comparison between tomograms simulated for (**a**,**b**) perpendicular polarisation and (**c**,**d**) parallel polarisation for a circular agglomeration of cylindrical scatterers with radius *a* = 0.5 *μ*m. (**a**,**c**) are calculated with the MAXWELL-solver, while (**b**,**d**) are calculated with the FDTD-based model. The values are displayed on a non-logarithmic scale and normalized to 1. With the MAXWELL-code, $N=80$ wave numbers and ${N}_{y}=61$ scan positions have been calculated with a cut-off value of $\varepsilon ={10}^{-8}$ for the spectrum. With the FDTD code, $N=2048$ wave numbers and ${N}_{y}=101$ scan positions have been calculated. The grid resolution is $\tfrac{{\lambda }_{0}}{13}$ for the MAXWELL-solver and $\tfrac{{\lambda }_{0}}{30}$ for the FDTD-code. The displayed values in (**a**,**c**) are the normalized values obtained with equation (16). The integrated errors are 0.085 for both perpendicular and parallel polarisation, indicating again good agreement between the simulations.

**Figure 8:**
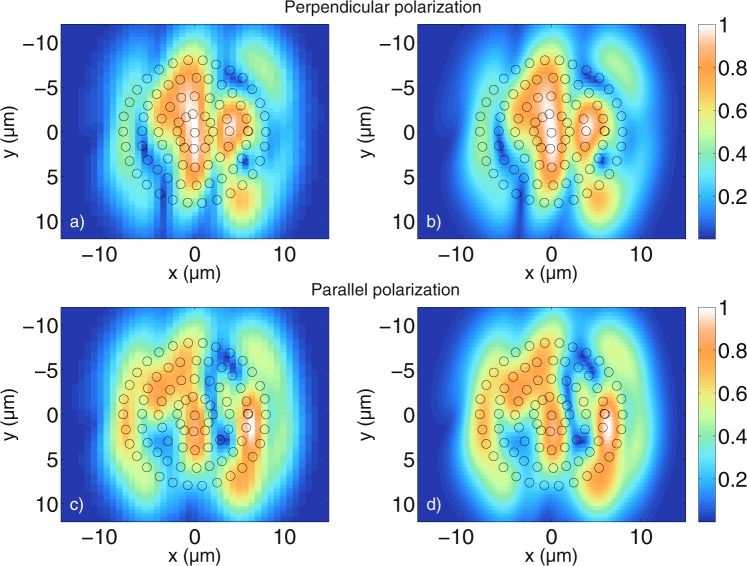
Comparison between tomograms simulated for (**a**,**b**) perpendicular polarisation and (**c**,**d**) parallel polarisation for a circular agglomeration of cylindrical scatterers shifted away from their original positions which are displayed in Fig. 7. The cylinder radius is *a* = 0.5 *μ*m and the values obtained with equation (16) are normalized to 1. (**a**,**c**) Are calculated with the MAXWELL-solver, while (**b**,**d**) are calculated with the FDTD algorithm. The simulation parameters are the same as those used for Fig. 7. The integrated error is 0.025 and 0.035 for perpendicular and parallel polarisation, respectively, indicating good agreement.

It has been shown that the calculated cylinder tomograms do not reveal the physical surface of the scatterers. In order to investigate whether the general shape of a distribution of multiple cylindrical scatterers can be imaged, many cylinders with a radius of 0.5 *μ*m and a refractive index of *n*_*cyl*_ = 1.42 are distributed within a circular area in the numerical grid of the MAXWELL-solver. Note that we do not expect to be able to resolve the individual cylindrical scatterers due to their size, but the total size of the circular area is larger than the resolution limit of the optical setup. The refractive index of the outer medium is again *n*_*med*_ = 1.35. In Fig. 7, the tomograms of 83 cylinders that are distributed in a symmetric manner, indicated by the black circles overlapping with the calculated tomograms, are shown. Figure 7(a) shows the resulting tomogram, calculated with the MAXWELL-solver for perpendicular polarisation, while Fig. 7(b) shows the tomogram calculated with the FDTD algorithm. Figure 7(c,d) show in the same way the results for parallel polarisation. It can be seen that in contrast to the tomograms of single cylinders from the previous sections, the results for many cylinders are more sensitive to the polarisation of the incident light, as has been observed previously^36^. Furthermore, as expected, the individual cylinders cannot be resolved by the given OCT system, however, the general outer contour of the round shape is visible. The shape of the simulated tomograms is complex due to the many interactions of the light with the cylindrical scatterers. There is good qualitative agreement between the results obtained with the MAXWELL-solver and the results obtained with the FDTD-based model. The influence of the individual scatterers is investigated by randomly shifting their positions from the original positions shown in Fig. 7. This was done by generating a random number for each of the *x*- and *y*-directions, and for each cylinder, resulting in the cylinders being shifted by a minimum of 0.06 nm and a maximum of 230 nm in *x*-direction and by a minimum of 14 nm and a maximum of 192 nm in *y*-direction. The maximum overall shift is 253 nm and the average overall shift is 88 nm. Note that cylinders were not prevented from overlapping, however, this does not occur frequently. Figure 8 shows the resulting tomograms calculated for both polarisations with the MAXWELL-solver and the FDTD-based model just like in Fig. 7.

**Figure 9:**
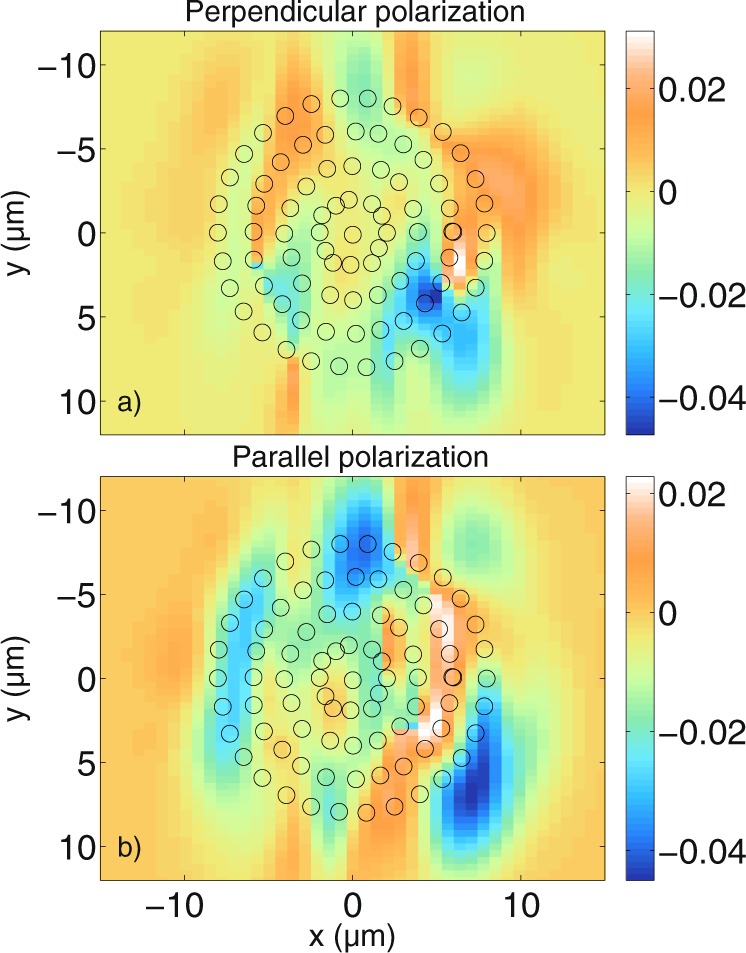
Absolute error of the data shown in Fig. 8. The absolute error is about 0.04 at maximum for both perpendicular (**a**) and parallel (**b**) polarisation. The absolute error is calculated as *I*_*MAXWELL*_ − *I*_*FDTD*_ where *I*_*MAXWELL*_ are the MAXWELL data and *I*_*FDTD*_ are the FDTD data.

**Figure 10:**
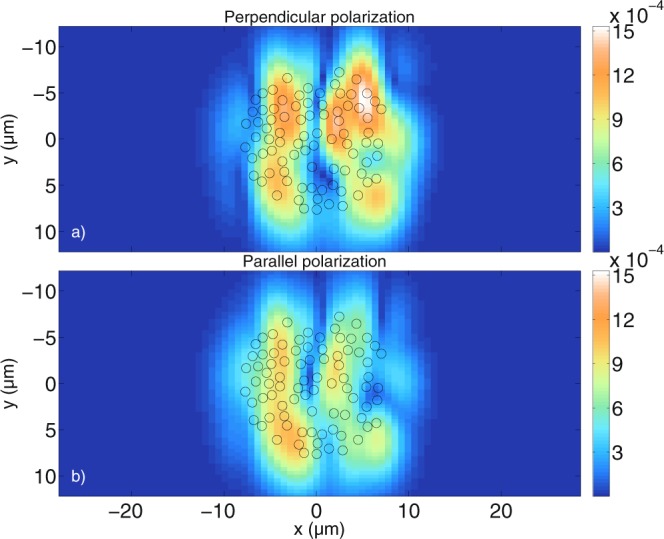
Tomograms for perpendicular (**a**) and parallel (**b**) polarisation of the incident light for randomly arranged cylindrical scatterers within a circular area. For this simulation, $N=80$ wavelengths and ${N}_{y}=61$ scan positions have been calculated with $\varepsilon ={10}^{-8}$. The grid resolution used is about $\tfrac{{\lambda }_{0}}{24}$. It can be seen that the results for perpendicular and parallel polarisation differ.

Figure 8(a,b) show the MAXWELL-solver and the FDTD-based results for perpendicular polarisation, while 8(c,d) show the MAXWELL-solver and the FDTD-based results for parallel polarisation, both showing good qualitative agreement. It can be seen that by shifting the cylinder positions slightly away from the symmetry position, one obtains a very different structure compared to the tomograms in Fig. 7. In particular, the tomograms obtained for randomly shifted cylinders begin to resemble a speckle pattern. Just like in Fig. 7, the results for the two polarisations differ considerably. It can be seen that for the case of perpendicular polarisation, the lateral bar-shaped area of highest intensity in Fig. 7 is distorted due to the shift of the scatterers, while for the case of parallel polarisation, the bar structure cannot be identfied with certainty in Fig. 8. The outline of the outer curvature of the scatterers is still visible. The performed simulations show that the positions of the scatterers in the given simulations have a large impact on the resulting tomograms. Besides the integrated error, the absolute error is calculated to show that the results obtained with the MAXWELL-solver and the FDTD-based model agree quantitatively as well as qualitatively. The absolute errors *I*_*MAXWELL*_ − *I*_*FDTD*_ for all points in Fig. 8 are plotted in Fig. 9. Figure 8(b,d) have a higher resolution and are scaled down to have the same number of points as Fig. 8(a,c), respectively. Figure 9(a,b) show that the absolute error between the tomograms is about 0.04. One has to keep in mind that due to the limited number of wave numbers calculated with the MAXWELL-solver, signals belonging to greater depths reappear at low distances in Fig. 8(a,c), inducing an additional error (see section “Maximum displayable depth”). All in all, the quantitative agreement is good. Having validated the tomograms, many different cylinder setups have been investigated and it would exceed the scope of this paper to display all simulations. For example, Fig. 10 shows tomograms for another realization of randomly arranged cylindrical scatterers calculated with the MAXWELL-solver for the perpendicular (10 a)) and the parallel field components (10 b)). The difference between the two polarisations is again clearly visible. The intensities obtained with equation (16) are not normalized in Fig. 10, but they are divided by the intensity from the reference arm calculated as ${\int }_{{\omega }_{{\min }}}^{{\omega }_{{\max }}}\,|{\alpha }_{ref}(\omega ){|}^{2}d\omega$. This means that the detected intensities are displayed with respect to the case where the sample is a second mirror at the focus position, with properties similar to the reference mirror. Therefore, the intensities of the following figures can be compared to each other. Comparing many tomograms for randomly distributed cylindrical scatterers reveals that small changes in the cylinder position have a large impact on the tomogram structure. Furthermore, as can be seen in Fig. 10, the positions of highest intensity do not simply coincide with the highest or lowest local density of the scatterers. Instead, all the simulated tomograms exhibit non-trivial structures that are related to the interference of the partial light paths through the scatterers and back to the detection fiber.

### Intensity and surface orientation

**Figure 11:**
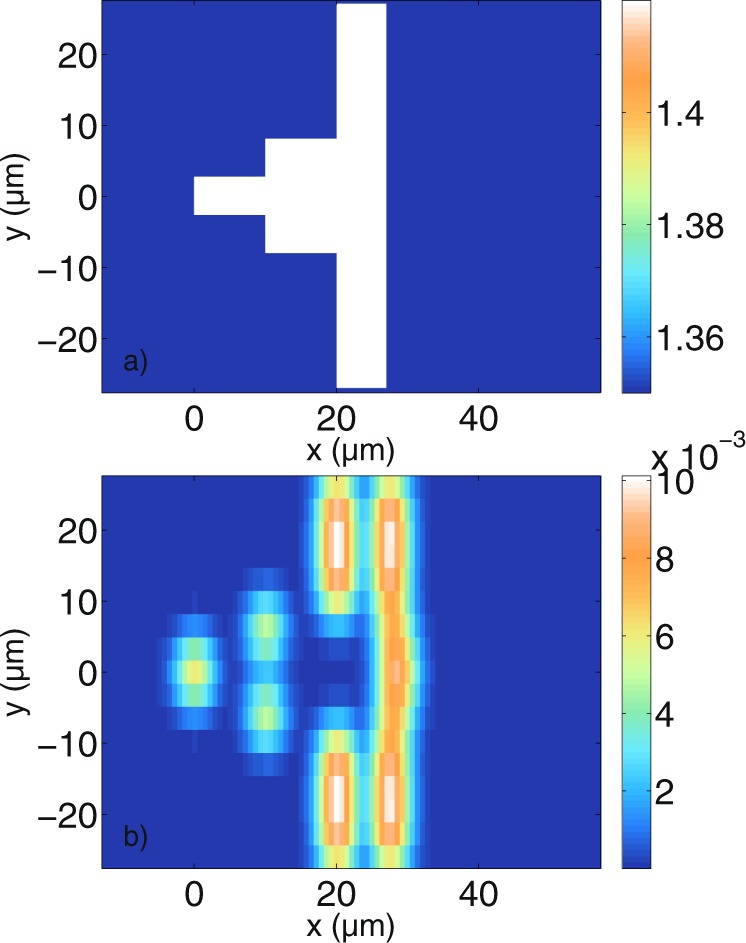
Grid with refractive indices (**a**) and tomogram for perpendicular polarisation (**b**) of a structure with several plateaus. With the given axial and lateral resolution, the boundaries can be identified in the tomogram. For this simulation, $N=160$ wavelengths and ${N}_{y}=17$ scan positions have been used. Furthermore, $\varepsilon ={10}^{-8}$ and the grid resolution used is $\tfrac{{\lambda }_{0}}{26}$.

The simulations from the previous sections show that the calculated tomograms do not, in general, accurately represent the surfaces of the scatterers. In the case where a plane surface is oriented perpendicularly to the incident beam, one would expect the signals in the tomogram to correspond more clearly to the given geometry. In Fig. 11, data for a ziggurat-shaped structure with different plateaus is shown. Figure 11(a) shows the refractive index distribution of the scatterer geometry while Fig. 11(b) shows the resulting tomogram for perpendicular polarisation. The tomogram for parallel polarisation is not shown here since it is similar. Again, the displayed values are calculated with equation (16) and divided by the intensity coupled into the detection fiber from the reference arm. One can see that with the given lateral and axial resolution, the plateaus can be distinguished. Furthermore, the surfaces with a larger lateral extension have a higher intensity in the tomogram since more light is coupled back into the detection fiber. The highest intensity is not located at *x* = 0 *μ*m as one might expect (compare the structure of the incident beam in Fig. 2). Signals stemming from the second boundary between scatterer and medium have a longer optical pathway back to the detection fiber. For this reason, these signals appear at a greater depths. The longest delay is found in the middle of the tomogram, at *y* ≈ 0, where a bump can be seen in the backreflection signal that is traveling through the medium.

### Simulation of tubules in dentin

**Figure 12:**
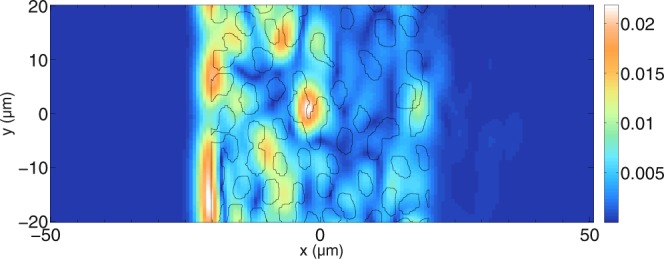
Simulated tomogram of a dentin slab for perpendicular polarisation. The positions of the tubules are indicated by black lines. Note that since the tubule sizes are below the resolution of the OCT system, we do not expect perfect correlation between sample geometry and the OCT image. For this simulation, $N=160$ wavelengths and ${N}_{y}=101$ scan positions have been calculated. The tomogram is displayed on a non-logarithmic scale. Furthermore, $\varepsilon ={10}^{-8}$ and the grid resolution used is $\tfrac{{\lambda }_{0}}{22}$. The mean free path for perpendicular polarisation inside the slab is about 7.0 *μ*m and one can see that the sample-induced scattering aberrates the focussed beam, first shown in Fig. 2 for a refractive index of the medium of 1.35, so that the focal region cannot be identified anymore.

Having presented several basic scatterer configurations in order to investigate OCT imaging with Maxwell’s equations, more realistic scatterer configurations for water-filled tubules in a dentin slab are now shown. Since the previous sections have shown that OCT does not always reveal the true physical surfaces of individual scatterers, it is interesting to see whether OCT images of dentin slabs show the actual position of the tubules. The dentin slab with the tubules varies less along the *z*-direction compared to that in the *x*-*y*-plane, so that the developed two-dimensional OCT model may be used. The dentin slab model is based on a light microscopy image presented in^68^. The image is interpolated and based on its grey-scale values, a refractive index of 1.33 is assumed for the water-filled tubules and the surrounding medium is assigned a refractive index of 1.52 for dentin. The background medium is assumed to have a refractive index of 1.52 everywhere so that the surface reflection of a boundary air-dentin is supressed. For all simulations, only the perpendicular component is shown since parallel polarisation yields similar results. The intensities obtained with equation (16) are divided by the reference arm intensity. In order to investigate the influence of the tubules on the tomogram, different concentrations are simulated. The lateral extension of the scanned area (40.0 *μ*m) is smaller than the lateral width of the grid (69.3 *μ*m) so that edge effects are reduced. Figure 12 shows the tomogram of a simulated dentin slab with the original concentration. The black lines show the real positions of the tubules. Note that since the outer medium has a higher refractive index than the water-filled tubules, one would expect light from greater depths that travels through many tubules to have the tendency to appear in front of the corresponding black lines. It can be seen in Fig. 12 that the first tubules, giving a signal from their boundary facing the incident beam, are visible in the tomogram. The two water-filled tubules close to each other at *y* ≈ 8 *μ*m cannot be distinguished due to the lateral resolution. The tubules at greater depths cannot be identified in the tomogram. Assuming that the tubules can be approximated as cylindrical scatterers, the mean free path length in the slab model with dimensions *L*_*x*_ and *L*_*y*_ is calculated as $L=\frac{\pi {a}^{2}}{{C}_{sca}f}$ with the concentration factor $f=\frac{{N}_{cyl}\pi {a}^{2}}{{L}_{x}{L}_{y}}$. *C*_*sca*_ is the scattering coefficent for infinitely long cylinders as described by Bohren and Huffman^54^. For ${N}_{cyl}\approx 122$ scatterers, *L*_*x*_ = 40.4 *μ*m, *L*_*y*_ = 69.3 *μ*m, and for an incident wavelength of $\lambda =\frac{1300}{1.52}\,{\rm{nm}}$, the mean free path length is 7.0 *μ*m when cylindrical scatterers with a radius of *a* = 1 *μ*m are assumed. This shows that most of the light undergoes multiple scattering, causing signals to appear at positions that are not easily linked to the positions of the tubules.

**Figure 13:**
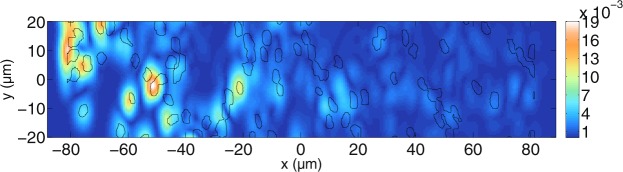
Simulated tomogram of a dentin slab for perpendicular polarisation. $N=280$ wavelengths and ${N}_{y}=101$ scan positions have been calculated. Furthermore, $\varepsilon ={10}^{-8}$ and the grid resolution used is about $\tfrac{{\lambda }_{0}}{13.5}$. The mean free path for perpendicular polarisation inside the slab is about 24.8 *μ*m, meaning that the focused beam cannot be identified anymore due to the scattering medium.

**Figure 14:**
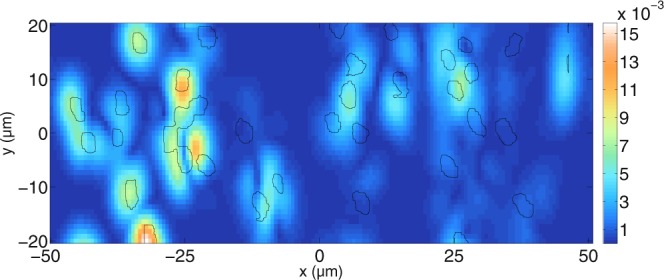
Simulated tomogram of a dentin slab for perpendicular polarisation. $N=160$ wavelengths and ${N}_{y}=45$ scan positions have been calculated. Furthermore, $\varepsilon ={10}^{-8}$ and the grid resolution used is about $\tfrac{{\lambda }_{0}}{13.5}$. The mean free path for perpendicular polarisation inside the slab is about 38.1 *μ*m, meaning that the quality of the beam focus is decreased at high depths inside the medium.

In order to alleviate this effect, the concentration of tubules is decreased. Figure 13 shows results for another simulation for ${N}_{cyl}\approx 127$ tubules in an area of *L*_*x*_ × *L*_*y*_ = 162.5 × 63.7 *μ*m^2^. Over the first half of the depth coordinate it can be seen that most signals in the tomograms can be linked to the positions of the tubules. With increasing depth, the correlation with the tubule positions decreases. It can also be seen that the intensity of the tomogram decreases with depth. The mean free path length is *L* = 24.8 *μ*m for the perpendicular polarisation shown in Fig. 13. More signals correlate with the tubule positions after having decreased the concentration. In order to further investigate the effect, the concentration of tubules is decreased further in Fig. 14.

Figure 14 shows the resulting tomogram for perpendicular polarisation. Again, the black lines show the positions of the water-filled tubules. The mean free path is now 38.1 *μ*m. It can be seen that more correlation between the actual positions of the tubules is achieved compared to Fig. 13. Note that the signal in the lower-left corner of Fig. 14 is not an artifact, but it stems from a tubule located just outside the scanning area.

**Figure 15:**
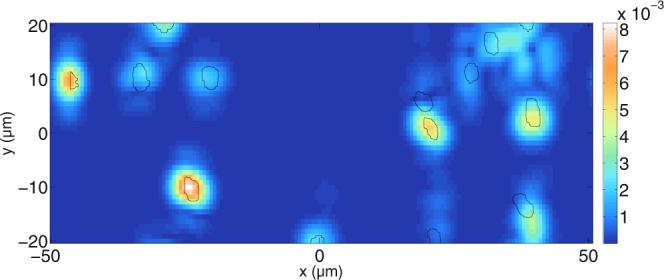
Simulated tomogram of a dentin slab for perpendicular polarisation. The simulation parameters are the same as those in Fig. 14. The mean free path for perpendicular polarisation inside the slab is about 77.9 *μ*m, meaning that the focus of the beam is preserved inside the medium.

**Figure 16:**
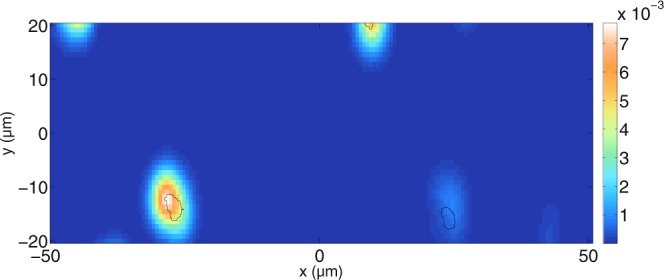
Simulated tomogram of a dentin slab for perpendicular polarisation. The simulation parameters are the same as those in Fig. 14. The mean free path for perpendicular polarisation inside the slab is about 224.1 *μ*m, meaning that the focused beam propagates nearly undistorted through the medium.

Reducing the scatterer concentration further shows the location of all tubules in the tomogram. This is shown in Fig. 15. Here, the mean free path is 77.9 *μ*m and all tubules can be identified. However, at greater depths, some artifacts are visible where no scatterers are located. Therefore, the scatterer concentration is reduced further in Fig. 16. The resulting mean free path is 224.1 *μ*m. In Fig. 16, all tubules can be identified and there are no artifacts present. The signals on top and bottom belong again to scatterers that are outside the scanned area. Therefore, one can conclude, as expected, that lower concentrations of tubules improve the quality of the tomograms so that the identification of tubule positions is less prone to errors.

## Conclusions

A two-dimensional algorithm for the simulation of OCT images was implemented and validated with an independent FDTD-based model. An expression for the maximum visible depth was derived and simulated tomograms of several scatterer geometries were investigated. Tomograms of single cylindrical scatterers were calculated and the origin of the individual signals was discussed. Tomograms of multiple cylindrical scatterers, of plane boundaries and of dentin models were presented as well. The simulated tomograms showed that care must be taken when interpreting OCT images. The smooth round surface of the considered single cylindrical scatterers was not visible in the tomograms. Furthermore, it was shown that in dentin slabs, artifacts can be mistaken for the position of scatterers, depending on the concentration of the water-filled tubules inside the dentin. Tomograms of many cylindrical scatterers yielded complex images with patterns that were sensitive to small shifts in the cylinder positions which showed that in order to understand the formed tomogram, the precise location of the scatterers must be known. The individual cylinder positions could not be detected, but the general outline of the outer contour of the scatterers could be traced. In tomograms of a ziggurat-shaped scatterer, the boundaries were much more readily identified. In short, by computing tomograms for various scattering geometries, the correlation between OCT tomogram and sample structure has been investigated successfully for several cases, showing that the tomograms do not accurately represent the surface of the scatterers. The simulations suggest that in order to understand image formation in OCT, both the wave properties of light and the properties and positions of the scattering structures must be taken into account.

## Supplementary information

Supplementary information

## Data Availability

The datasets generated during the current study are available from the corresponding author on reasonable request.
