# Supplementary material for: Two-dimensional simulation of optical coherence tomography images
Source: Sci Rep. 2019 Aug 21;9:12189. doi: 10.1038/s41598-019-48498-2 (PMC6704163; doi:10.1038/s41598-019-48498-2)
Supplement: Supplementary file 1 — Supplementary information [file 41598_2019_48498_MOESM1_ESM.pdf]

Supplementary information

Two-dimensional simulation of optical coherence tomography images

Thomas Brenner[1,*], **Peter Munro**[2], **Benjamin Krüger**[1], **and Alwin Kienle**[1]

[1]Institut für Lasertechnologien in der Medizin und Meßtechnik an der Universität Ulm, Ulm, 89081, Germany
[2]University College London, Department of Medical Physics and Biomedical Engineering, London, WC1E 6BT, UK
[*]thomas.brenner@ilm-ulm.de

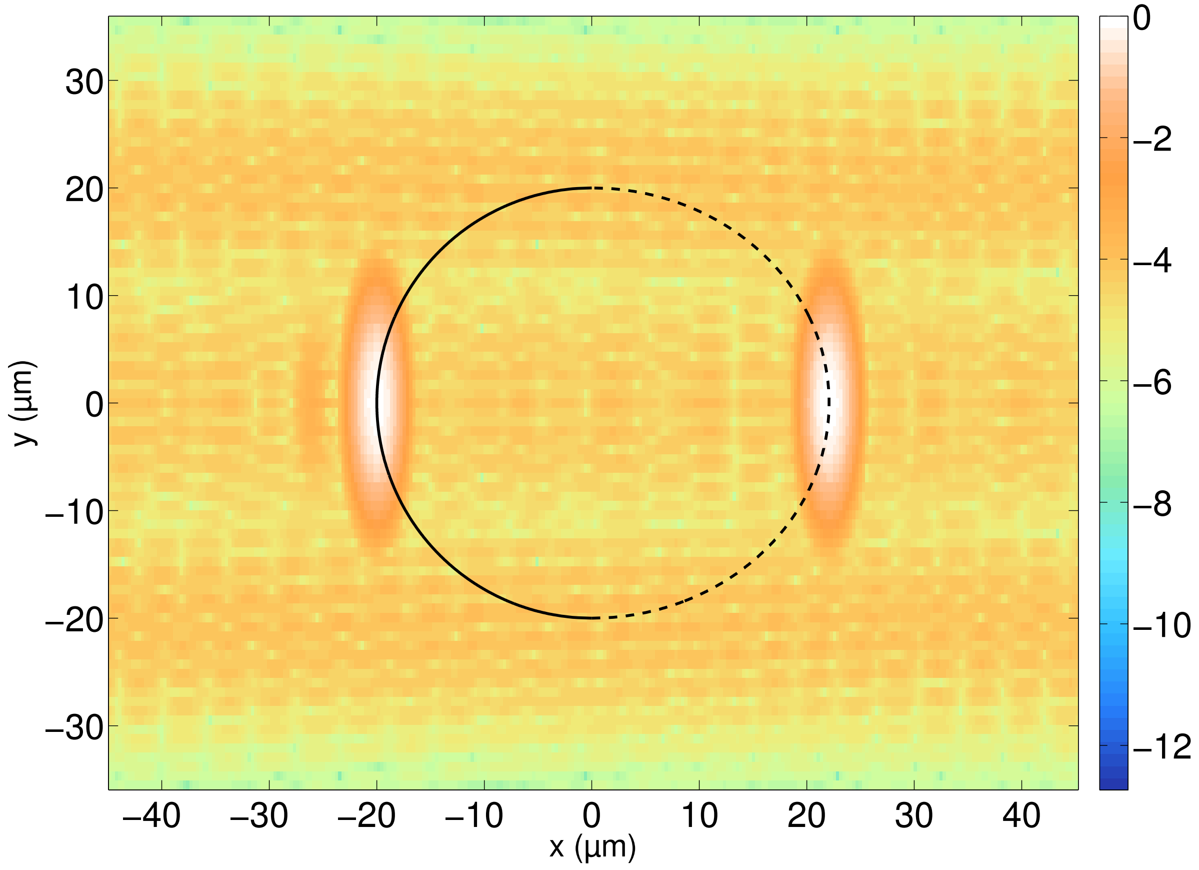

Supplementary Figure 1. The full tomogram, calculated with cylinder theory, for a cylindrical scatterer with $a = 20\ \mu$m radius and refractive index $n_{cyl} = 1.42$ in a medium of 1.35 for a central wavelength of $\lambda_0 = 845$ nm, plotted on a logarithmic scale for perpendicular polarisation. Here, no surface waves are visible. The black line indicates the cylinder side facing the scanning beam while the dotted line shows the distortion of the other side due to the refractive index of the cylinder.
